# Reliability of Markerless 2D Video Analysis for Calculating Gait Joint Angles in Uncontrolled Environments

**DOI:** 10.3390/s26196229

**Published:** 2026-09-30

**Authors:** Sagrario Pérez-de la Cruz, Francisco Molina-Rueda, Pilar Fernández-González

**Affiliations:** 1 Department of Nursery, Physiotherapy and Medicine, University of Almería, 04120 Almería, Spain; 2 Department of Physical Therapy, Occupational Therapy, Rehabilitation and Physical Medicine, Faculty of Health Sciences, Rey Juan Carlos University, 28922 Madrid, Spain; francisco.molina@urjc.es (F.M.-R.); pilar.fernandez@urjc.es (P.F.-G.)

**Keywords:** biomechanics, kinematics, Kinovea, motor control, reliability, walking

## Abstract

**Highlights:**

**What are the main findings?**

- Core lower limb sagittal kinematics were successfully assessed in ecological field settings using 2D markerless video tracking.
- Cross-sectional gait evaluation demonstrated moderate-to-good inter-rater reliability, whereas between-session intra-rater reliability was poor (particularly at the hip and ankle).

**What are the implications of the main findings?**

- Lower between-session intra-rater reliability reflects evaluator subjectivity in repeated manual landmark digitization over time without physical skin markers.
- The established Minimal Detectable Change (MDC) values provide measurement-error estimates for repeated video ratings in healthy samples, defining the baseline variability inherent to visual tracking without skin markers.

**Abstract:**

**Background:** Traditional 3D gait analysis constrains natural movement variability due to artificial laboratory settings; thus, this study evaluated the intra- and inter-rater reliability of a low-cost, 2D markerless smartphone video protocol for assessing lower limb sagittal kinematics in an unconstrained environment. **Methods:** Forty-five healthy young adults walked at a self-selected speed in a non-laboratory corridor, where sagittal hip, knee, and ankle angles were recorded at 60 fps and analyzed using Kinovea^®^ software by two independent raters, with one rater repeating measurements seven days later. **Results:** Inter-rater reliability ranged from moderate to good across most joint variables (ICC = 0.628–0.789), except for the ankle during loading response, which showed poor consistency (ICC = 0.381). Between-session intra-rater reliability was generally lower, ranging from poor to moderate (ICC = 0.278–0.580), with the lowest values observed at the hip (ICC = 0.278) and ankle (ICC = 0.391) during loading response. Minimal Detectable Change (MDC95) values ranged from 7.07° to 8.95° for inter-rater assessments and from 7.40° to 16.55° for intra-rater assessments, with Bland–Altman analysis displaying wide limits of agreement longitudinally. **Conclusions:** In conclusion, while prior observer training standardizes criteria for immediate cross-sectional evaluations, the protocol’s high sensitivity to measurement variability and unconstrained motor execution limits its clinical application for longitudinal tracking across different sessions. Between-session intra-rater variability reflects evaluator subjectivity in manual frame selection and landmark digitization over time when no physical markers are present, rather than biological gait fluctuations.

## 1. Introduction

Gait analysis is a fundamental tool for evaluating human locomotion, enabling the identification of biomechanical alterations, longitudinal monitoring of therapeutic progress, and objective assessment of clinical intervention efficacy [1]. Although three-dimensional (3D) motion capture systems represent the established benchmark due to their precision [2], their high cost, spatial demands, complex calibration, and operational requirements hinder their routine clinical integration [2,3,4].

To overcome these barriers, two-dimensional (2D) video analysis has emerged as an accessible alternative. Evidence indicates that when standardized acquisition protocols are implemented, 2D lower limb joint kinematics correlate strongly with 3D motion capture [5]. Furthermore, the proliferation of smartphone cameras and video analysis software—such as Kinovea^®^, a free open-source software—has fostered the integration of low-cost tools into practice [6,7]. In gait, Kinovea^®^ has demonstrated good validity and reliability during initial contact [3] and trunk tilt assessment [8]. Beyond gait, it has been applied in running, jumps, valgus, and dynamic assessment [9,10,11,12], and even as a secondary benchmark to validate wearable sensors [13].

However, 2D captures cannot quantify out-of-plane transverse movements [3,14]. Moreover, measurement fidelity depends on landmark identification, camera setup, lighting, and evaluator experience [15,16]. While laboratory standardization minimizes noise [6], clinical utility depends on performance within unconstrained healthcare settings exhibiting greater ecological validity. To date, evidence regarding markerless Kinovea^®^ reliability outside laboratory boundaries remains sparse.

Transitioning to unconstrained environments addresses a foundational motor control paradigm: ecological validity versus motor variability. Standardized laboratories force participants into artificial replication tasks, suppressing the natural, functional trial-to-trial kinematic variability of locomotion [17]. Evaluating gait in unconstrained settings captures this organic motor variability, which conventional protocols obscure. However, markerless tools must demonstrate that they can operate in field conditions while distinguishing true clinical changes from intrinsic biological noise across separate sessions and raters.

Therefore, it is hypothesized that while inter-rater reliability will remain moderate-to-good due to standardized digital tracking criteria during simultaneous evaluation, between-session intra-rater reliability across repeated rating sessions will reveal greater variability associated with visual landmark estimation over time on unmarked digital recordings.

## 2. Materials and Methods

### 2.1. Participants

A convenience sample of healthy volunteers was recruited from the National Police Academy through informative open calls. Inclusion criteria comprised being over 18 years of age, engaging in regular recreational physical activity (excluding competitive or professional athletics), presenting no neuromuscular, musculoskeletal, or osteoarticular conditions altering gait or posture, and walking without orthoses or mobility aids. Individuals reporting lower extremity injuries within six months prior to testing were excluded.

The study protocol received formal approval from the local Bioethics Committee (UALBIO2023/062). All participants received detailed verbal and written explanations of the procedures and signed an institutional informed consent form prior to data collection.

### 2.2. Instrumentation

Gait trials were recorded using a Samsung Galaxy A54 5G smartphone camera (Samsung Electronics Co., Ltd. Suwon, South Korea- Republic of Korea-) equipped with a 6.4-inch Super AMOLED FHD+ screen (1080 × 2340 pixels), recording native video at 60 frames per second at Full HD resolution (1080p). The device was stabilized on a tripod positioned laterally at a fixed perpendicular distance of 2.5 m from the midpoint of the walking corridor, at a camera height of 1.0 m.

Kinematic digitization was conducted using Kinovea^®^ software (version 0.9.5), a free, open-source 2D motion analysis platform that incorporates perspective correction algorithms based on spatial image calibration. Kinovea^®^ has been previously validated for measuring articular ranges of motion, jump performance, physical conditioning exercises, and gait parameters [10,12,18,19].

### 2.3. Experimental Procedure

Data acquisition took place between March and May 2024 at the National Police Academy facilities through a collaboration between the University of Almería (Spain) and Rey Juan Carlos University (Spain). Demographic characteristics—including age, biological sex, body weight, standing height, lower limb dominance, and dominant limb length—were collected via a standardized questionnaire.

For spatial image calibration, two high-contrast floor markers were placed exactly 2.0 m apart along the central walking path parallel to the camera vector. Participants performed a 10-min warm-up walk along the unobstructed corridor to acclimate to the environment and establish their comfortable, self-selected walking speed. Participants wore form-fitting sportswear (e.g., short tights or fitted underwear, exposing the upper torso in male participants) to eliminate loose fabric interference and maximize visual identification of anatomical landmarks.

No automated marker tracking algorithms or retroreflective skin markers were utilized for digital angle calculation. Although participants wore passive foot markers for a separate concurrent 3D study, evaluators completely disregarded these markers during digitization, placing kinematic tools strictly via direct visual estimation of key anatomical landmarks. Each participant performed two walking trials separated by a brief rest period. For each participant, one representative gait cycle from the second walking trial of the dominant lower limb was selected for analysis to eliminate initial acceleration effects and ensure steady-state locomotion.

### 2.4. Kinematic Digitization

Digital video files were evaluated independently by two trained physical therapists experienced in clinical motion analysis. Before analyzing study trials, both observers completed a structured calibration training program supervised by an expert in biomechanics to standardize frame identification and landmark placement criteria. Observer 1 evaluated all video trials at Session 1 and repeated the full analysis seven days later (Session 2) to determine intra-rater reliability across time. To prevent recall bias during the intra-rater evaluation at Session 2 (7 days later), all video files were anonymized, renamed using random identification codes, and presented in a randomized sequence. Observer 1 was completely blinded to the initial Session 1 measurements. Observer 2 evaluated the Session 1 videos independently to establish inter-rater reliability.

Two distinct functional events within the gait cycle were operationalized for frame selection:

1. Loading response (Foot-Flat): operationalized as the specific video frame immediately following initial contact in which the plantar surface of the stance foot establishes complete flat contact with the floor, marking the termination of loading response.
2. Mid-Swing (Contralateral Limb Passage): operationalized as the video frame where the swinging lower extremity passes directly parallel to the stance shank, representing maximum lower limb acceleration and clearance.

**Figure 1:**
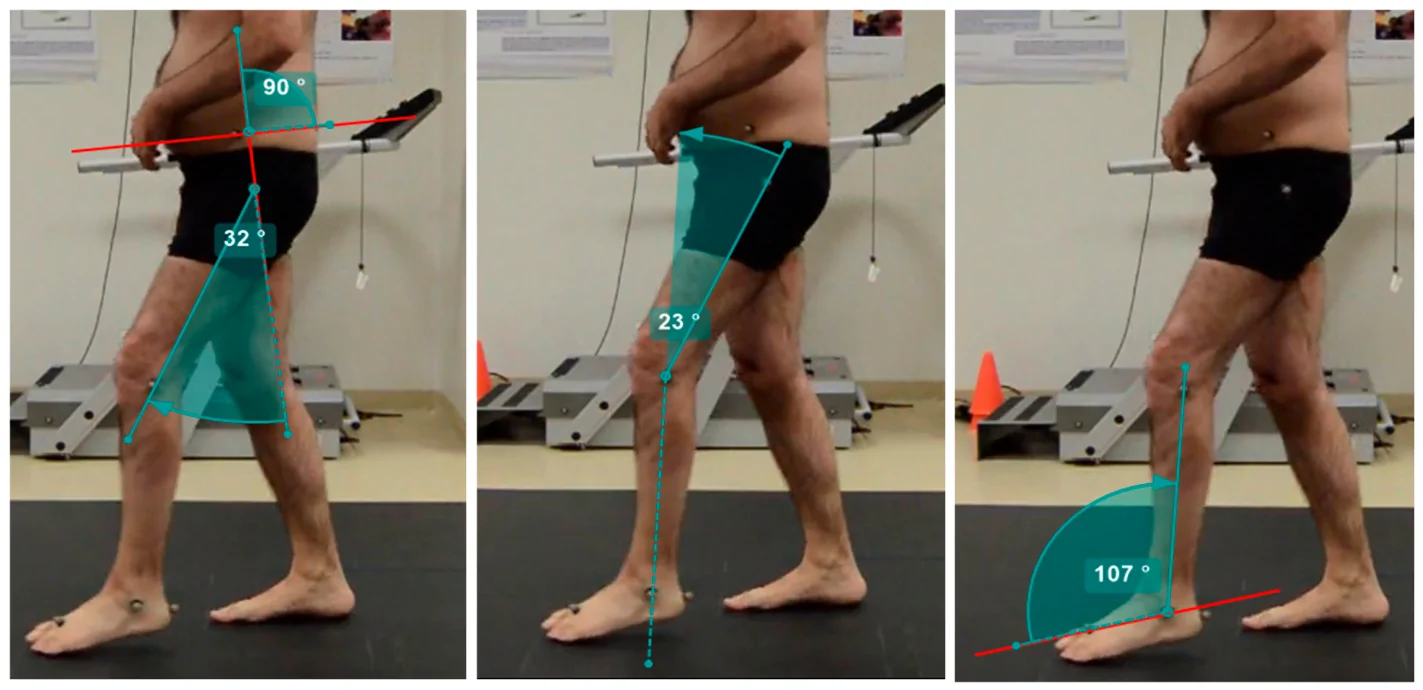
Tracking of hip, knee, and ankle angles using Kinovea^®^ software.

Joint angle conventions were standardized as follows [Figure 1]:

For the hip angle, a constructed pelvic reference line (shown in red) connects the Anterior Superior Iliac Spine (ASIS/EAS) and Posterior Superior Iliac Spine (PSIS/EPS). A perpendicular bisector passing through the greater trochanter is erected at 90° to this pelvic line. The hip angle vertex is positioned at the greater trochanter, with one ray extending toward the lateral femoral condyle and the second ray aligned with the perpendicular pelvic reference line.

Methodological note: the spherical markers visible on the subject’s foot correspond to the simultaneous calibration of an external 3D motion capture system. These markers were completely ignored by the raters, who conducted the 2D kinematic analysis relying exclusively on visual estimation of anatomical landmarks.

- Hip angle: determined relative to a constructed pelvic reference line. A primary reference line was drawn connecting the anterior superior iliac spine (ASIS) and posterior superior iliac spine (PSIS). A perpendicular bisector passing through the greater trochanter was erected at 90° to the pelvic line. The hip angle vertex was placed at the greater trochanter; one ray aligned with the lateral femoral condyle and the second ray aligned with the perpendicular pelvic line. Neutral standing was designated as 0°, with positive values indicating hip flexion.
- Knee angle: quantified using the angle tool positioned with its vertex at the lateral femoral condyle. One ray extended to the greater trochanter and the other to the lateral malleolus. The supplementary angle was calculated, where 0° represented full knee extension and positive values indicated knee flexion.
- Ankle angle: formed by constructing a reference line parallel to the lateral border of the foot through the lateral malleolus. The angle vertex was placed at the lateral malleolus, with one ray directed along the lateral foot line toward the anterior foot and the second ray pointing toward the lateral femoral condyle. The raw angle provided by Kinovea^®^ (θraw) was converted to a signed anatomical ankle angle using the formula: θankle = θraw − 90°, where 0° corresponds to neutral standing (90° relative position), positive values denote dorsiflexion, and negative values denote plantarflexion.

### 2.5. Sample Size Calculation

Sample size estimation was conducted following Walter et al. [20] based on the primary variable: knee joint angle during mid-swing, which demonstrates the largest sagittal plane excursion. Setting a minimally acceptable Intraclass Correlation Coefficient (*ICC*_0_) of 0.60, an expected target coefficient (*ICC*_1_) of 0.80, *α* = 0.05, and statistical power at 80% (*β* = 0.20), a minimum requirement of 39 participants was established. Accounting for a potential 10% data attrition or video artifact rate, a final cohort of 45 participants was recruited.

### 2.6. Statistical Analysis

Statistical processing was performed using SPSS Statistics version 22.0 (IBM Corp., Armonk, NY, USA). Descriptive statistics (mean ± standard deviation for continuous variables; frequencies and percentages for categorical data) were computed. Normality was confirmed using Shapiro–Wilk testing.

Relative reliability was quantified using Intraclass Correlation Coefficients (ICC) with 95% confidence intervals (95% CI) according to Koo and Li [21]:

- Inter-rater reliability: calculated between Observer 1 and Observer 2 for Session 1 using a two-way random-effects, absolute agreement, single-measures model [ICC (2,1)].
- Intra-rater reliability: calculated for Observer 1 between Session 1 and Session 2 (7-day interval) using a two-way mixed-effects, absolute-agreement, single-rating model [ICC (3,1)]. Following Koo and Li [21], a two-way mixed-effects model was selected for intra-rater reliability because the single evaluator (Observer 1) was fixed and represented the specific clinical rater of interest, while absolute agreement was specified to account for systematic differences across measurement sessions.

Absolute agreement and systematic bias were evaluated using Bland–Altman analysis with 95% limits of agreement (LoA = d ± 1.96 · SDdiff), where d represents the mean difference between measurements [22]. Absolute measurement precision was quantified via the Standard Error of Measurement (SEM) calculated using the pooled standard deviation (SDpooled):

SEM = SDpooled · √(1 − ICC),

where SDpooled was calculated as:

SDpooled = √((SD1^2^ + SD2^2^)/2)

True clinical change thresholds were established by calculating the Minimal Detectable Change at a 95% confidence level:

$${\mathrm{MDC}}_{95}=1.96\cdot \sqrt{2}\cdot \mathrm{SEM}$$

Statistical significance was set at *p* < 0.05.

## 3. Results

Forty-five healthy subjects (25 men [55.6%], 20 women [44.4%]) successfully completed the study protocol without dropping out or generating corrupted video files. Demographics were mean age 30.40 ± 5.43 years, body weight 74.31 ± 12.15 kg, height 176.13 ± 8.35 cm, BMI 23.89 ± 2.45 kg/m^2^, and dominant lower limb length 93.91 ± 4.38 cm. Right-side limb dominance was present in 41 participants (91.1%) and left-side dominance in 4 participants (8.9%). Gait speed of the participants fell within a comfortable range of 1.3 to 1.4 m/s.

Across most evaluated joint variables, inter-rater reliability demonstrated higher point estimates than intra-rater reliability between sessions (Table 1). Bland–Altman scatter plots confirmed narrower limits of agreement for inter-observer comparisons, reflecting reduced measurement dispersion between raters evaluating the same video session compared to a single rater evaluating sessions separated by a 7-day interval.

Systematic bias between raters remained minimal across all joints, with absolute mean differences ranging between 0.32° and 1.71°, confirming the absence of systematic over- or under-estimation between observers. Intra-rater systematic bias across sessions was slightly higher, reaching a maximum mean difference of 2.22° for hip loading response. Absolute measurement precision across variables exhibited SEM values between 2.55° and 5.97°, translating to MDC95 values between 7.07° and 16.55°.

For the hip joint, intra-rater reliability over time was poor during loading response (ICC = 0.278; 95% CI: −0.002 to 0.521; *p* = 0.027; SEM = 5.97°; MDC95 = 16.55°) and poor-to-moderate during mid-swing (ICC = 0.435; 95% CI: 0.173 to 0.642; *p* = 0.001; SEM = 4.92°; MDC95 = 13.64°). Limits of agreement for intra-rater hip measurements were wide (ULoA = 18.67°, LLoA = −14.24° for loading response). Conversely, inter-rater reliability for the hip achieved moderate agreement during loading response (ICC = 0.674; 95% CI: 0.462 to 0.810; *p* < 0.001; SEM = 3.23°; MDC95 = 8.95°) and good agreement during mid-swing (ICC = 0.789; 95% CI: 0.631 to 0.882; *p* < 0.001; SEM = 2.68°; MDC95 = 7.43°).

For the knee joint, intra-rater reliability was moderate during loading response (ICC = 0.580; 95% CI: 0.349 to 0.744; *p* < 0.001; SEM = 3.20°; MDC95 = 8.87°) and poor-to-moderate during mid-swing (ICC = 0.424; 95% CI: 0.153 to 0.636; *p* = 0.002; SEM = 3.74°; MDC95 = 10.37°). Inter-rater reliability for the knee demonstrated good agreement during loading response (ICC = 0.750; 95% CI: 0.588 to 0.854; *p* < 0.001; SEM = 2.66°; MDC95 = 7.37°) and moderate agreement during mid-swing (ICC = 0.662; 95% CI: 0.461 to 0.798; *p* < 0.001; SEM = 2.55°; MDC95 = 7.07°).

**Figure 2:**
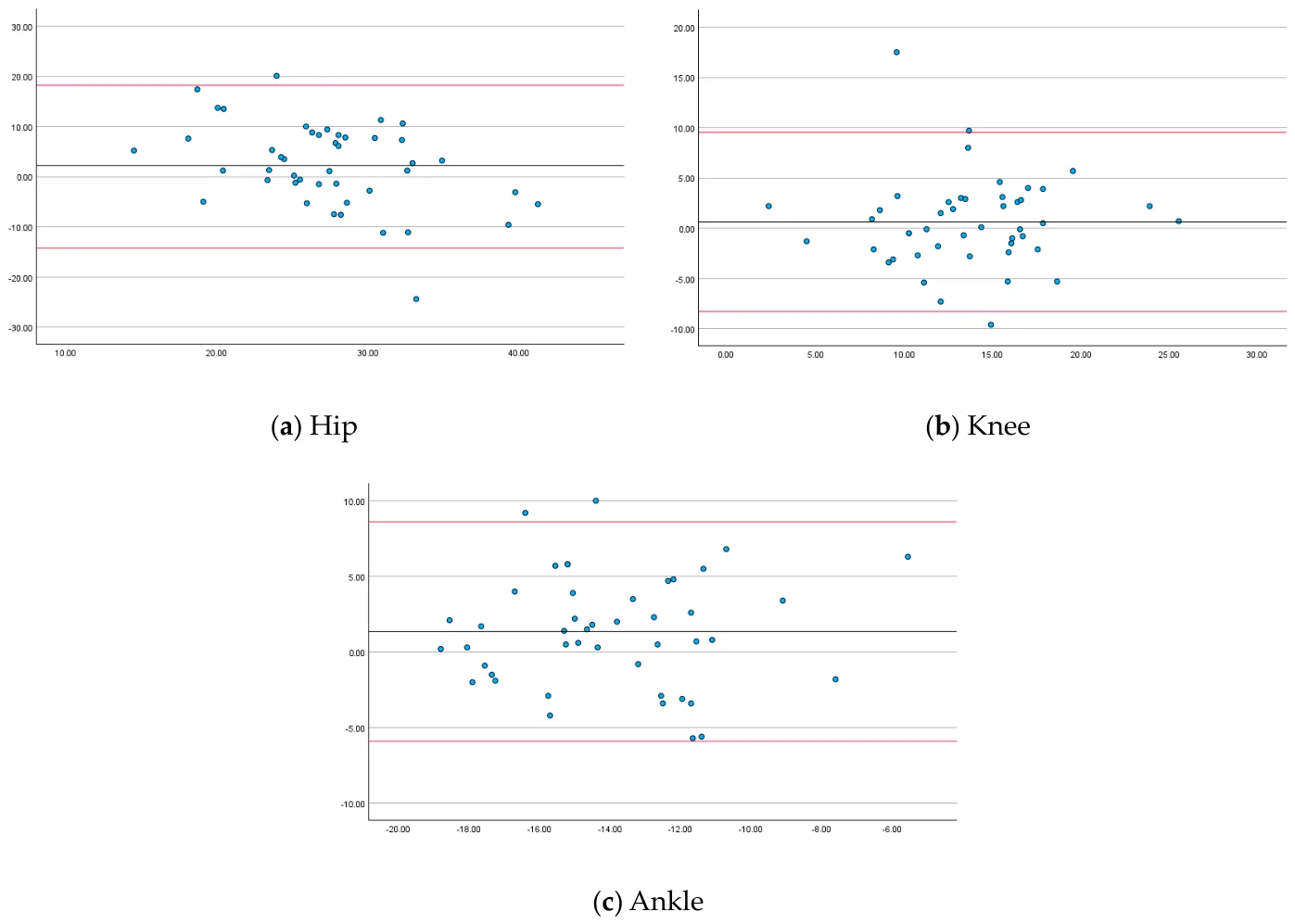
Bland–Altman plots comparing between-session measurement results (for observer 1) of hip (**a**), knee (**b**), and ankle (**c**) angles during the loading response phase. For each parameter, the bias (black line) and limits of agreement (red lines) are shown. The mean is represented on the X-axis and the between-session difference (mean of differences) on the Y-axis (mean difference ± 1.96 SD).

**Figure 3:**
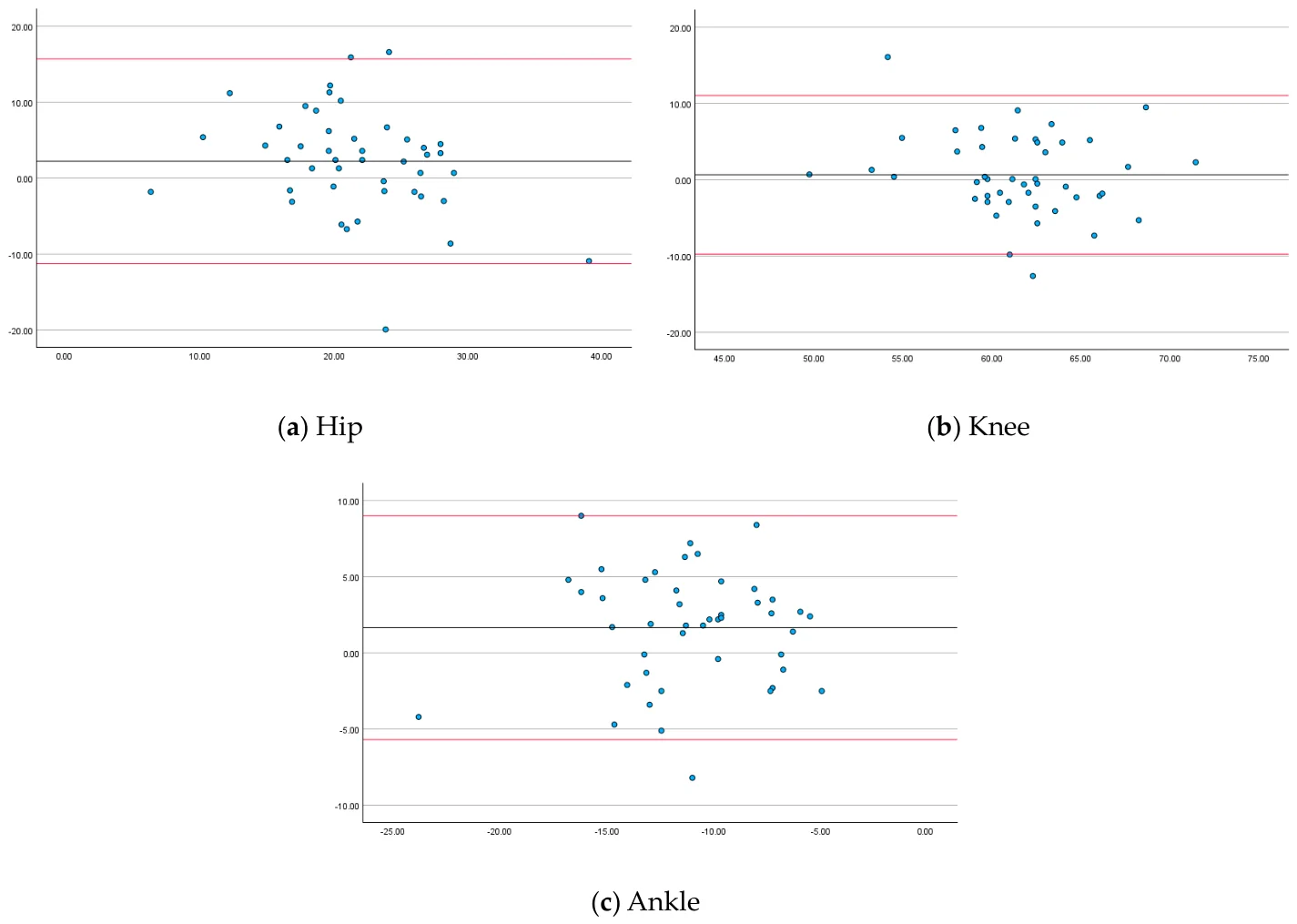
Bland–Altman plots comparing between-session measurement results (for observer 1) of hip (**a**), knee (**b**), and ankle (**c**) angles during the mid-swing phase. For each parameter, the bias (black line) and limits of agreement (red lines) are shown. The mean is represented on the X-axis and the between-session difference (mean of differences) on the Y-axis (mean difference ± 1.96 SD).

**Figure 4:**
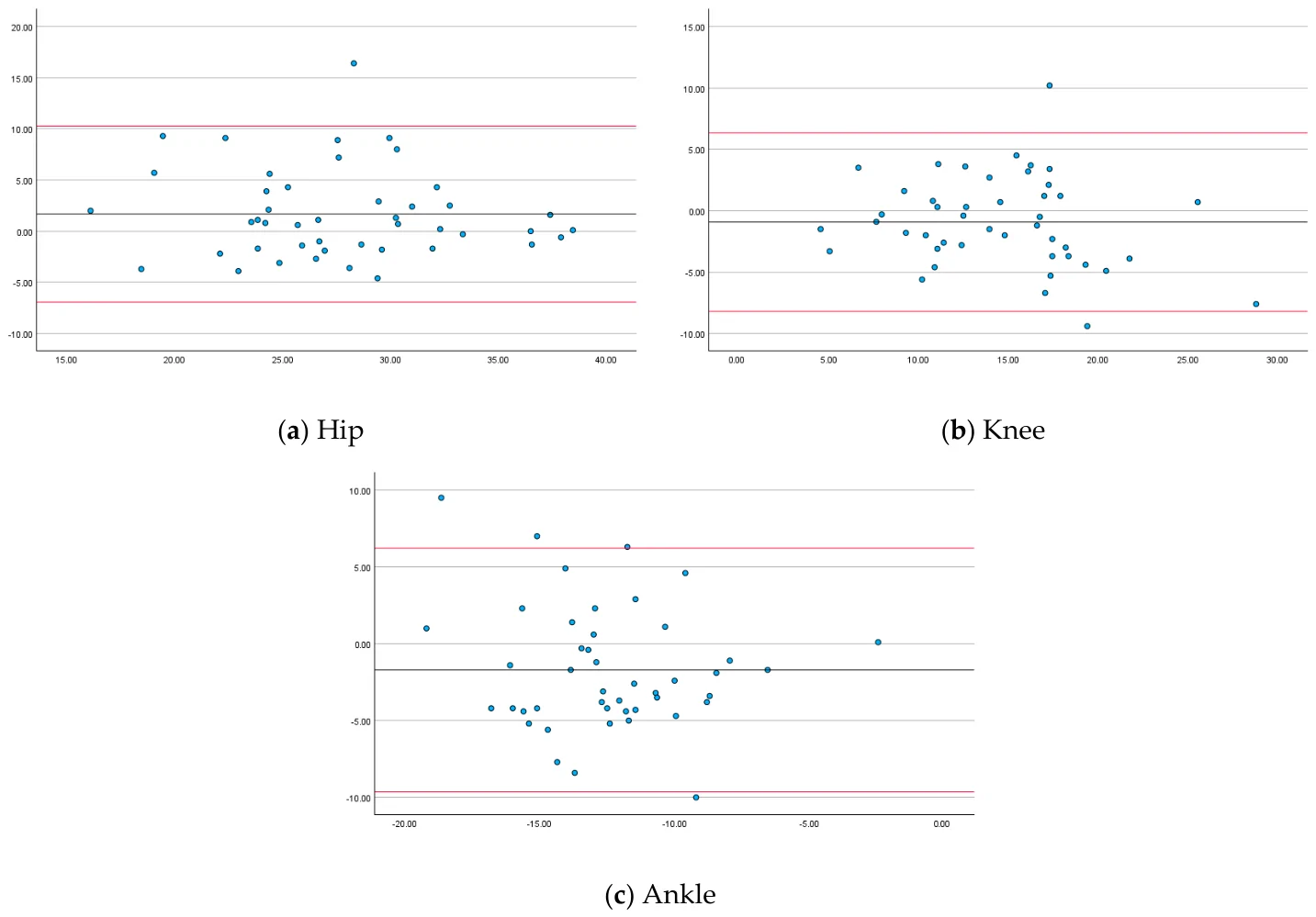
Bland–Altman plots comparing inter-rater results of hip (**a**), knee (**b**), and ankle (**c**) angles during the loading response phase. For each parameter, the bias (black line) and limits of agreement (red lines) are shown. The mean is represented on the X-axis and the inter-rater difference (mean of differences) on the Y-axis (mean difference ± 1.96 SD).

**Figure 5:**
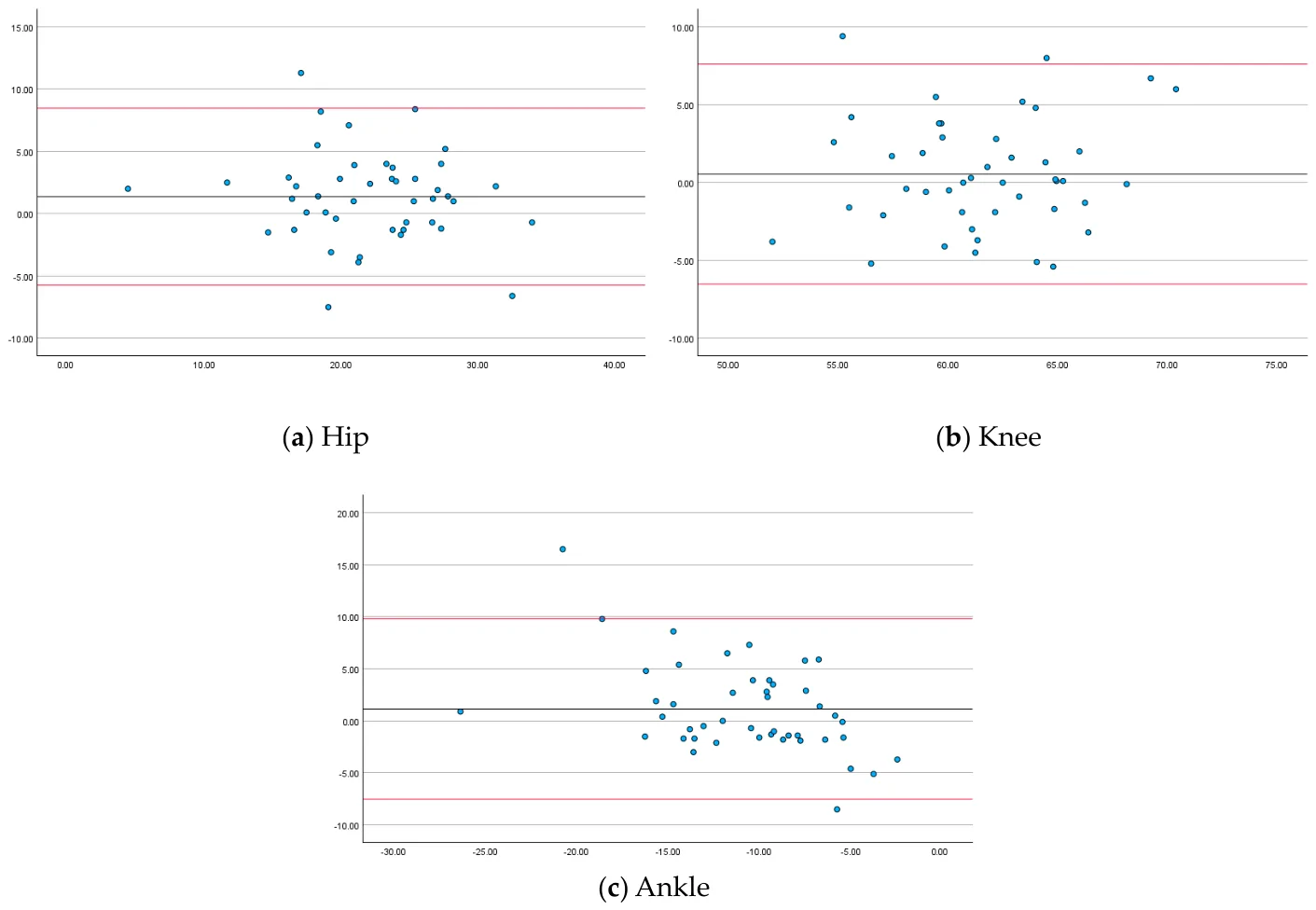
Bland–Altman plots comparing inter-rater results of hip (**a**), knee (**b**), and ankle (**c**) angles during the mid-swing phase. For each parameter, the bias (black line) and limits of agreement (red lines) are shown. The mean is represented on the X-axis and the inter-rater difference (mean of differences) on the Y-axis (mean difference ± 1.96 SD).

For the ankle joint, intra-rater reliability was poor during loading response (ICC = 0.391; 95% CI: 0.123 to 0.608; *p* = 0.002; SEM = 2.67°; MDC95 = 7.40°) and moderate during mid-swing (ICC = 0.548; 95% CI: 0.287 to 0.729; *p* < 0.001; SEM = 2.77°; MDC95 = 7.68°). Inter-rater reliability similarly showed poor consistency during loading response (ICC = 0.381; 95% CI: 0.109 to 0.602; *p* = 0.002; SEM = 2.94°; MDC95 = 8.15°) but reached moderate agreement during mid-swing (ICC = 0.628; 95% CI: 0.414 to 0.776; *p* < 0.001; SEM = 3.16°; MDC95 = 8.76°). Figure 2, Figure 3, Figure 4 and Figure 5 show Bland–Altman plots comparing the results of cross-session measurements (for observer 1) of hip (a), knee (b) and ankle (c) angles during the load response phase (Figure 2), during the mid-swing phase (Figure 3), during the load-response phase (Figure 4) and during the mid-swing phase (Figure 5).

## 4. Discussion

This study evaluated the intra- and inter-rater reliability of markerless 2D video analysis using Kinovea^®^ software for measuring lower limb joint angles during the loading response and mid-swing phases of gait in unconstrained, real-world corridor settings. The main findings indicate that reliability varies substantially depending on the specific joint, gait phase, and observational condition. Overall, knee joint angles demonstrated the highest consistency, whereas hip and ankle joint kinematics displayed moderate-to-poor reliability, particularly when assessed across separate testing sessions.

A primary finding of this investigation is that while same-day inter-rater agreement ranged from moderate to good, test-retest intra-rater reliability over a 7-day interval was consistently lower. Rather than invalidating markerless 2D video analysis, these results establish critical operational boundaries for clinical transfer. In unconstrained field environments—characterized by standard flooring, street clothing, ambient lighting, and the absence of fixed physical skin markers—minor deviations in camera positioning, perspective, or individual walking line between days introduce non-systematic variance. Consequently, while Kinovea^®^ provides a dependable tool for cross-sectional assessments (e.g., multiple clinicians assessing a patient concurrently or evaluating performance within a single session), practitioners must exercise caution when utilizing this markerless setup for longitudinal monitoring over time, as unconstrained environmental variance may obscure or artificially inflate longitudinal clinical changes.

The reduced intra-rater reliability observed for the hip and ankle during loading response reflects human measurement variability rather than biological gait fluctuations or software calculation errors. Because the same video recordings were re-analyzed seven days later without physical markers attached to the participants’ skin, the observed differences stem directly from evaluator subjectivity when manually identifying ambiguous anatomical landmarks (such as the greater trochanter or lateral malleolus) and selecting specific video frames across separate rating sessions. Without superficial physical markers to serve as fixed visual anchors, minor variations in the evaluator’s visual estimation of bony landmarks across days introduce non-systematic rating noise, particularly in complex multi-axial joints like the hip and ankle.

This measurement error interpretation is supported by our inter-rater data. Because both observers evaluated the exact same video cycles during Session 1 using standardized calibration criteria, inter-observer agreement was notably higher than test-retest intra-rater agreement over time. Thus, markerless 2D video analysis functions reliably for concurrent cross-sectional evaluations, whereas the lower intra-rater values highlight the temporal subjectivity of human visual digitizing when analyzing unmarked video recordings across different sessions.

Regarding absolute reliability, comparing absolute error metrics against relative ICC values is necessary to establish clinical utility. While high ICCs can mask constant angular offsets between raters, our absolute error parameters demonstrate that systematic bias remained low across all conditions (<2.22°). However, broader limits of agreement and elevated MDC_95_ values (reaching 5.84° for hip loading response) highlight random variance arising from visual landmark estimation without physical markers. This random variance defines the baseline error margin that clinical progress must exceed to represent genuine functional improvement.

Our intra-rater reliability values are lower than those reported by Fernández-González et al. [3], who documented excellent intra-observer reliability (*ICC* > 0.85) for hip, knee, and ankle angles during initial contact. However, their protocol was conducted under controlled laboratory conditions utilizing skin markers and evaluated only initial contact. In contrast, our protocol evaluated loading response and mid-swing—gait events that require dynamic frame selection within continuous movement—without physical markers and in an unconstrained corridor. This methodological divergence aligns with recent evidence demonstrating that video analysis reliability declines when moving from controlled laboratory setups to unstandardized field environments [16].

Across analyzed joints, the knee exhibited the most consistent intra- and inter-rater performance during loading response. This robustness stems from the clear visibility of knee anatomical landmarks in the sagittal plane and the substantial angular excursion of the joint, consistent with running studies showing high reliability for knee joint tracking [15]. Conversely, the hip displayed lower reliability across phases. This finding agrees with prior literature emphasizing that 2D hip angle calculations are vulnerable to pelvic tilt, lumbar position, and unrecorded transverse pelvic rotation [3,14].

Ankle kinematics yielded intermediate consistency, achieving moderate inter-rater reliability during mid-swing but declining during loading response. Accurately defining the foot’s lateral border and lateral malleolus alignment in low-contrast video frames introduces visual ambiguity, especially during rapid footstrike transitions. Similar constraints regarding ankle landmark digitization and visual angle perspective have been documented in field-based video studies of running footstrike patterns [15,16].

A methodologically notable result was that inter-rater reliability exceeded intra-rater reliability across most variables, particularly during loading response. Psychometrically, this indicates that supervised observer calibration successfully unified digital tracking criteria and frame identification between independent raters for simultaneous analysis. However, the drop in intra-rater reliability across the 7-day interval demonstrates that without physical markers, temporal subjectivity influences visual landmark estimation over time. Clinicians naturally vary their visual interpolation of bony landmarks through clothing across separate days, introducing temporal observer bias that limits longitudinal tracking utility.

From a clinical application standpoint, the MDC_95_ thresholds reported here establish clear decision-making parameters. Because between-session MDC_95_ values reached 2.5° to 5.8°, patients must demonstrate kinematic changes exceeding these specific cut-offs before clinicians can confidently attribute improvements to therapeutic interventions rather than environmental or measurement noise.

Contextualizing our findings within broader Kinovea^®^ literature highlights task-dependent reliability. High test-retest reliability has been established for cervical and wrist range of motion [10,18], vertical jumps [11,23], and dynamic functional movements [12]. However, those clinical tasks involve planar, highly repeatable movements under stationary conditions. Locomotion is a complex dynamic task where environmental variations introduce multi-planar rotations that degrade single-camera 2D tracking stability [15,16]. Nevertheless, our findings align with recent real-world validation studies. Suansomchit et al. [13] utilized Kinovea^®^ to validate wearable sensors during wheelchair maneuvers, reporting moderate-to-good agreement under non-laboratory conditions and concluding that modest reliability reductions outside laboratories do not negate clinical utility.

Several limitations must be acknowledged. First, conducting trials in an unconstrained corridor precluded fixed laboratory calibration frames. Parallax error—caused by subtle shifts in perpendicular camera alignment as participants moved along the corridor—remains an intrinsic constraint that contributed to test-retest variation. Second, because the protocol was strictly markerless, evaluators estimated joint centers visually on screen without physical skin markers. While inter-rater consistency remained solid, the absence of fixed markers introduced variability when replicating landmark coordinates across days.

Third, 2D single-camera video cannot record transverse or frontal plane movements, exposing sagittal calculations to out-of-plane crosstalk [3,14]. Fourth, commercial 60 fps smartphone recording may introduce a 16.6 ms frame-sampling window, potentially missing peak joint events during rapid stance phase transitions. Fifth, evaluating healthy young adults limits direct generalizability to clinical populations exhibiting severe gait pathology. Finally, post-hoc analysis indicated that while the sample size (*n* = 45) was sufficiently powered (80%) for primary variables expecting moderate agreement (*ICC* ≥ 0.60), secondary parameters exhibiting low ICCs (e.g., hip loading response) were underpowered to establish precise reliability estimates.

Furthermore, absolute joint angle values obtained in this study exhibited systematic offsets compared to classical 3D optoelectronic normatives [1]. These offsets stem from estimating bony landmarks through clothing, camera parallax over extended corridors, and out-of-plane joint rotations projected onto a single 2D image plane [7,14,16]. Consequently, while markerless 2D video analysis is valuable for evaluating relative kinematic trends and gross movement shifts within individual subjects, absolute 2D angle values should not be directly equated with 3D motion capture norms.

Future research should evaluate markerless Kinovea^®^ protocols in clinical cohorts, explore artificial intelligence-assisted semi-automated landmark tracking, and assess combined setups incorporating low-cost inertial sensors to resolve multi-planar gait motion outside the laboratory.

## 5. Conclusions

Markerless 2D video analysis using Kinovea^®^ software in unconstrained environments provides an accessible, low-cost clinical tool; however, its measurement reliability varies depending on the evaluated joint and gait phase. While prior observer calibration successfully unifies criteria for single-session cross-sectional evaluations, the reliance on visual landmark estimation without physical skin markers introduces temporal rater subjectivity that limits its precision for longitudinal tracking across separate sessions. Clinicians and researchers should utilize this markerless 2D setup primarily for single-session cross-sectional evaluations, recognizing that the established Minimal Detectable Change (MDC95) values—ranging from 7.07° to 16.55°—represent measurement-error estimates for repeated rating procedures in healthy individuals rather than validated thresholds for clinical improvement.

## Figures and Tables

**Table 1:** Intra-rater and inter-rater reliability for the calculation of hip, knee, and ankle angles during the loading response and mid-swing phases of gait.

Outcome Measure	Phase/Side	Session 1 O1	Session 2 O1	Session O2	Type	ICC	95%CI	*p*	ULoA	LLoA	SEM	MDC
Hip angle (degrees)	LR	28.55 (5.46)	26.33 (8.31)	26.88 (5.85)	Intra	0.278	−0.002 to 0.521	0.027	18.67	−14.24	5.97	16.55
					Inter	0.674	0.462 to 0.810	<0.001	10.28	−6.93	3.23	8.95
	MS	22.78 (5.61)	20.54 (7.42)	21.41 (6.06)	Intra	0.435	0.173 to 0.642	0.001	15.70	−11.24	4.92	13.64
					Inter	0.789	0.631 to 0.882	<0.001	8.47	−5.74	2.68	7.43
Knee angle (degrees)	LR	14.06 (4.97)	13.43 (4.91)	14.98 (5.65)	Intra	0.580	0.349 to 0.744	<0.001	9.52	−8.27	3.20	8.87
					Inter	0.750	0.588 to 0.854	<0.001	6.35	−8.19	2.66	7.37
	MS	61.85 (4.53)	61.18 (5.31)	61.3 (4.23)	Intra	0.424	0.153 to 0.636	0.002	11.07	−9.74	3.74	10.37
					Inter	0.662	0.461 to 0.798	<0.001	7.62	−6.51	2.55	7.07
Ankle angle (degrees)	LR	−13.27 (3.48)	−14.62 (3.36)	−11.56 (3.97)	Intra	0.391	0.123 to 0.608	0.002	8.60	−5.90	2.67	7.40
					Inter	0.381	0.109 to 0.602	0.002	6.22	−9.63	2.94	8.15
	MS	−10.26 (4.17)	−11.92 (4.08)	−11.40 (6.03)	Intra	0.548	0.287 to 0.729	<0.001	9.01	−5.69	2.77	7.68
					Inter	0.628	0.414 to 0.776	<0.001	9.80	−7.51	3.16	8.76

Values for sessions 1 and 2 are expressed as mean and standard deviation. O1 is observer 1; O2 is observer 2. LR is loading response; MS is mid-swing. ICC is Intraclass Correlation Coefficient; 95% CI is 95% Confidence Interval; p is statistical significance value; SEM is Standard Error of Measurement (calculated using SDpooled); MDC is Minimal Detectable Change (MDC95); ULoA is Upper Limit of Agreement; LLoA is Lower Limit of Agreement. ICC (2,1) was used for inter-rater reliability and ICC (3,1) for intra-rater reliability.

## Data Availability

The datasets presented in this article are not readily available because the data are part of an ongoing study, and due to technical and time limitations. Requests to Access the datasets should be directed to spd205@ual.es.
